# Distill: a suite of web servers for the prediction of one-, two- and three-dimensional structural features of proteins

**DOI:** 10.1186/1471-2105-7-402

**Published:** 2006-09-05

**Authors:** Davide Baú, Alberto JM Martin, Catherine Mooney, Alessandro Vullo, Ian Walsh, Gianluca Pollastri

**Affiliations:** 1 School of Computer Science and Informatics, University College Dublin, Belfield, Dublin 4, Ireland

## Abstract

**Background:**

We describe Distill, a suite of servers for the prediction of protein structural features: secondary structure; relative solvent accessibility; contact density; backbone structural motifs; residue contact maps at 6, 8 and 12 Angstrom; coarse protein topology. The servers are based on large-scale ensembles of recursive neural networks and trained on large, up-to-date, non-redundant subsets of the Protein Data Bank. Together with structural feature predictions, Distill includes a server for prediction of C_*α *_traces for short proteins (up to 200 amino acids).

**Results:**

The servers are state-of-the-art, with secondary structure predicted correctly for nearly 80% of residues (currently the top performance on EVA), 2-class solvent accessibility nearly 80% correct, and contact maps exceeding 50% precision on the top non-diagonal contacts. A preliminary implementation of the predictor of protein C_*α *_traces featured among the top 20 Novel Fold predictors at the last CASP6 experiment as group Distill (ID 0348). The majority of the servers, including the C_*α *_trace predictor, now take into account homology information from the PDB, when available, resulting in greatly improved reliability.

**Conclusion:**

All predictions are freely available through a simple joint web interface and the results are returned by email. In a single submission the user can send protein sequences for a total of up to 32k residues to all or a selection of the servers. Distill is accessible at the address: .

## Background

De novo prediction of protein three-dimensional structure from the primary sequence remains a fundamental and extraordinarily challenging problem. Many one-dimensional and two-dimensional structural features, i.e. structural properties of individual residues or of couples of residues in a protein, have long been identified as useful intermediate representations between the primary sequence and the full three-dimensional structure, which can be adopted as a stage towards the prediction of protein structure and function. For instance accurate secondary structure and solvent accessibility information have been shown to improve the sensitivity of fold recognition methods (e.g. [1,2]) and are at the core of most *ab initio *methods (e.g. see [3]) for the prediction of protein structure.

We have developed a number of predictors of structural features of proteins. Some of the features predicted are novel and highly informative (e.g. protein contact density [4], local structural motifs [5], multi-class coarse contact maps [6]), others are well established (secondary structure, solvent accessibility, residue contact maps) but predicted at state-of-the-art accuracy levels [4,7].

All methods are freely available through simple web interfaces, which allow the processing of medium- to large-scale jobs by any selection of the servers with only a small number of manual submissions.

## Implementation

### Algorithms

All one- and two-dimensional structural feature predictors are based on single- or dual-layer Recursive Neural Network architectures for Directed Acyclic Graphs (DAG RNNs) [8]. One-dimensional feature predictors (i.e. those mapping the primary sequence into a sequence of the same length) are based on 1D DAG RNNs [9], while two-dimensional feature predictors (where a property of pairs of residues or secondary structure elements is predicted) are based on 2D DAG RNNs [8,10], or combinations of the two [6]. All RNNs have *shortcut *connections to cut the length of paths between different inputs. This facilitates the transmission of long-range information, which is relevant to determine non-local structural properties such as the formation of *β*-sheets. In dual-layer RNNs, the second layer, or filter, incorporates long-range information directly (e.g. predicted secondary structure and solvent accessibility composition, averaged over multiple contiguous windows). In this way, information from up to 225 residues is taken into account when a final prediction is made. For a more detailed description of the models and training algorithms, see [4-7,11]. All systems adopt large-scale ensembles of predictors (40 or more models for each architecture), trained on large, non-redundant datasets extracted from the PDB [12].

The predictor of C_*α *_traces relies on a simple optimisation procedure, similar to that in [13], guided by a potential or pseudo-energy based on one- and two-dimensional feature predictions. The target of the optimisation is realising the C_*α *_trace which enforces the predicted features (e.g. presence of helices, presence or absence of contacts between residues) "best", while preserving some trivial properties such as realistic distances between neighbouring C_*α*_s and absence of clashes. The optimisation algorithm and potential are described in detail in [11].

### Data sets

All predictors of one-dimensional and two-dimensional features are trained on datasets extracted from the December 2003 25% pdb_select set [14]. We use the DSSP program [15] to assign target structural features and remove sequences for which DSSP does not produce an output due, for instance, to missing entries or format errors. After processing by DSSP, the set contains 2171 protein and 344,653 amino acids (set S2171).

Multiple sequence alignments for S2171 are extracted from the NR database as available on March 3 2004 containing over 1.4 million sequences. The database is first redundancy reduced at a 98% threshold, leading to a final 1.05 million sequences. The alignments are generated by three runs of PSI-BLAST [16]. Multiple alignment generation in online server operation is handled transparently and does not require user intervention.

The predictor of C_*α *_traces is benchmarked on a subset of the PDB available on April 5 2005 generated as follows: all proteins showing more than 22% sequence similarity to S2171 or shorter than 30 residues were excluded; the remaining set was redundancy reduced at a maximum 22% sequence similarity threshold; proteins longer than 200 residues were excluded. The final set contains 258 proteins (S258).

### Servers

**Figure 2:**
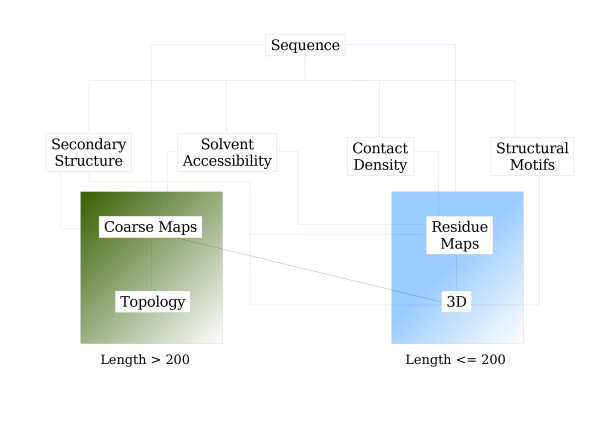
**Distill's flowchart**. Information flows from the top of the chart to the bottom.

The Distill suite of servers currently contains 7 predictors: 4 of one-dimensional features (Porter, Porter+, Pale Ale, BrownAle); 2 of two-dimensional features (XStout and XXStout); the predictor of C_*α *_traces (3Distill). The chart in Figure 2 represent the flow of information between the servers. Details about each server are provided below.

### Secondary structure

Porter [7] is a system for protein secondary structure prediction based on an ensemble of 1D DAG-RNNs. Porter is an evolution of the SSpro [17] server. Porter's improvements include: rich input coding (each residue is coded as a letter out of an alphabet of 25); output filtering and incorporation of predicted long-range information (225 residues are considered to yield the final predictions); large training sets (S2171); large-scale ensembling (45 models). Moreover, when this is available, homology information to structures in the PDB is provided as a further input to the system [18] (see results section).

#### Structural motifs

Porter+ [5] classifies each residue into one out of 14 local structural motifs. The motifs are built by applying multidimensional scaling and clustering to pair-wise angular distances between quadruplets of Φ – Ψ dihedral angle pairs collected from high-resolution protein structures [19]. Structural motif predictions are highly informative and provide a finer-resolution picture of a protein backbone than (and may be used to improve [5]) traditional 3-class secondary structure. The definition and one-letter code for the 14 structural motifs are provided in the help web page.

#### Relative solvent accessibility

PaleAle, a novel component of Distill, classifies each residue as being in one of 4 (approximately equally frequent) classes of solvent accessibility: B = completely buried (0–4% exposed); b = partly buried (4–25% exposed); e = partly exposed (25–50% exposed); E = completely exposed (more than 50% exposed). The architecture of PaleAle's classifier is an exact copy of Porter's (described above and in [7]). As in the case of Porter, when available, homology information to structures in the PDB is provided as a further input, yielding more accurate predictions [18] (see results section).

#### Contact density

BrownAle [4] is a system for the prediction of protein Contact Density. We define Contact Density as the Principal Eigenvector (PE) of a protein's residue contact map at 8Å, multiplied by the principal eigenvalue. Let *λ*(*C*) = {*λ *: *Cx *= *λx*} be the spectrum of *C *(where *C *is a protein's contact map, for whose definition see below), S
 MathType@MTEF@5@5@+=feaafiart1ev1aaatCvAUfKttLearuWrP9MDH5MBPbIqV92AaeXatLxBI9gBamrtHrhAL1wy0L2yHvtyaeHbnfgDOvwBHrxAJfwnaebbnrfifHhDYfgasaacH8akY=wiFfYdH8Gipec8Eeeu0xXdbba9frFj0=OqFfea0dXdd9vqai=hGuQ8kuc9pgc9s8qqaq=dirpe0xb9q8qiLsFr0=vr0=vr0dc8meaabaqaciaacaGaaeqabaWaaeGaeaaakeaaimaacqWFse=uaaa@3845@_*λ *_= {*x *: *Cx *= *λx*} the corresponding eigenspace and λ¯
 MathType@MTEF@5@5@+=feaafiart1ev1aaatCvAUfKttLearuWrP9MDH5MBPbIqV92AaeXatLxBI9gBaebbnrfifHhDYfgasaacH8akY=wiFfYdH8Gipec8Eeeu0xXdbba9frFj0=OqFfea0dXdd9vqai=hGuQ8kuc9pgc9s8qqaq=dirpe0xb9q8qiLsFr0=vr0=vr0dc8meaabaqaciaacaGaaeqabaqabeGadaaakeaaiiGacuWF7oaBgaqeaaaa@2E7F@ = max{*λ *∈ *λ*(*C*)} the largest eigenvalue of *C*. The principal eigenvector of *C*, x¯
 MathType@MTEF@5@5@+=feaafiart1ev1aaatCvAUfKttLearuWrP9MDH5MBPbIqV92AaeXatLxBI9gBaebbnrfifHhDYfgasaacH8akY=wiFfYdH8Gipec8Eeeu0xXdbba9frFj0=OqFfea0dXdd9vqai=hGuQ8kuc9pgc9s8qqaq=dirpe0xb9q8qiLsFr0=vr0=vr0dc8meaabaqaciaacaGaaeqabaqabeGadaaakeaacuWG4baEgaqeaaaa@2E3D@, is the eigenvector corresponding to λ¯
 MathType@MTEF@5@5@+=feaafiart1ev1aaatCvAUfKttLearuWrP9MDH5MBPbIqV92AaeXatLxBI9gBaebbnrfifHhDYfgasaacH8akY=wiFfYdH8Gipec8Eeeu0xXdbba9frFj0=OqFfea0dXdd9vqai=hGuQ8kuc9pgc9s8qqaq=dirpe0xb9q8qiLsFr0=vr0=vr0dc8meaabaqaciaacaGaaeqabaqabeGadaaakeaaiiGacuWF7oaBgaqeaaaa@2E7F@, i.e. such that *C*x¯
 MathType@MTEF@5@5@+=feaafiart1ev1aaatCvAUfKttLearuWrP9MDH5MBPbIqV92AaeXatLxBI9gBaebbnrfifHhDYfgasaacH8akY=wiFfYdH8Gipec8Eeeu0xXdbba9frFj0=OqFfea0dXdd9vqai=hGuQ8kuc9pgc9s8qqaq=dirpe0xb9q8qiLsFr0=vr0=vr0dc8meaabaqaciaacaGaaeqabaqabeGadaaakeaacuWG4baEgaqeaaaa@2E3D@ = λ¯
 MathType@MTEF@5@5@+=feaafiart1ev1aaatCvAUfKttLearuWrP9MDH5MBPbIqV92AaeXatLxBI9gBaebbnrfifHhDYfgasaacH8akY=wiFfYdH8Gipec8Eeeu0xXdbba9frFj0=OqFfea0dXdd9vqai=hGuQ8kuc9pgc9s8qqaq=dirpe0xb9q8qiLsFr0=vr0=vr0dc8meaabaqaciaacaGaaeqabaqabeGadaaakeaaiiGacuWF7oaBgaqeaaaa@2E7F@x¯
 MathType@MTEF@5@5@+=feaafiart1ev1aaatCvAUfKttLearuWrP9MDH5MBPbIqV92AaeXatLxBI9gBaebbnrfifHhDYfgasaacH8akY=wiFfYdH8Gipec8Eeeu0xXdbba9frFj0=OqFfea0dXdd9vqai=hGuQ8kuc9pgc9s8qqaq=dirpe0xb9q8qiLsFr0=vr0=vr0dc8meaabaqaciaacaGaaeqabaqabeGadaaakeaacuWG4baEgaqeaaaa@2E3D@. Thus, we define a protein's contact density as λ¯
 MathType@MTEF@5@5@+=feaafiart1ev1aaatCvAUfKttLearuWrP9MDH5MBPbIqV92AaeXatLxBI9gBaebbnrfifHhDYfgasaacH8akY=wiFfYdH8Gipec8Eeeu0xXdbba9frFj0=OqFfea0dXdd9vqai=hGuQ8kuc9pgc9s8qqaq=dirpe0xb9q8qiLsFr0=vr0=vr0dc8meaabaqaciaacaGaaeqabaqabeGadaaakeaaiiGacuWF7oaBgaqeaaaa@2E7F@x¯
 MathType@MTEF@5@5@+=feaafiart1ev1aaatCvAUfKttLearuWrP9MDH5MBPbIqV92AaeXatLxBI9gBaebbnrfifHhDYfgasaacH8akY=wiFfYdH8Gipec8Eeeu0xXdbba9frFj0=OqFfea0dXdd9vqai=hGuQ8kuc9pgc9s8qqaq=dirpe0xb9q8qiLsFr0=vr0=vr0dc8meaabaqaciaacaGaaeqabaqabeGadaaakeaacuWG4baEgaqeaaaa@2E3D@.

BrownAle predicts Contact Density in 4 classes. The class thresholds are assigned so that the classes are approximately equally numerous, as follows: N = very low contact density (0,0.04); n = medium-low contact density (0.04,0.18); c = medium-high contact density (0.18,0.54); C = very high contact density (greater than 0.54). BrownAle's architecture is an exact copy of Porter's (described above). Secondary Structure (by Porter) is fed as input into BrownAle, beside the primary sequence. Predicted Contact Density contributes significantly to improved residue contact map predictions [4], especially for long-ranged contacts.

#### Coarse contact maps and topologies

XStout [6] is a system for the prediction of protein coarse topologies. A protein is represented by a set of rigid rods associated with its secondary structure elements (*α*-helices and *β*-strands, as predicted by Porter). 4-class distance maps and 3-class angle maps between secondary structure elements are first predicted based on the primary sequence and on predicted secondary structure and solvent accessibility (by Porter and PaleAle), and coarse 3D folds of proteins are then assembled starting from these maps. 3D reconstruction is carried out by minimising a cost function taking the form of a purely geometrical potential. Coarse folds are only predicted when C_*α *_trace predictions are not available (for proteins longer than 200 residues), or not requested by the user.

#### Residue contact maps

XXStout [4] is a system for the prediction of protein residue contact maps. The contact map of a protein with *N *amino acids is a symmetric *N *× *N *matrix *C*, with elements *C*_*ij *_defined as:

Cij={1if amino acid i and j are in contact0otherwise     (1)
 MathType@MTEF@5@5@+=feaafiart1ev1aaatCvAUfKttLearuWrP9MDH5MBPbIqV92AaeXatLxBI9gBaebbnrfifHhDYfgasaacH8akY=wiFfYdH8Gipec8Eeeu0xXdbba9frFj0=OqFfea0dXdd9vqai=hGuQ8kuc9pgc9s8qqaq=dirpe0xb9q8qiLsFr0=vr0=vr0dc8meaabaqaciaacaGaaeqabaqabeGadaaakeaacqWGdbWqdaWgaaWcbaGaemyAaKMaemOAaOgabeaakiabg2da9maaceqabaqbaeaabiGaaaqaaiabigdaXaqaaiabbMgaPjabbAgaMjabbccaGiabbggaHjabb2gaTjabbMgaPjabb6gaUjabb+gaVjabbccaGiabbggaHjabbogaJjabbMgaPjabbsgaKjabbccaGiabbMgaPjabbccaGiabbggaHjabb6gaUjabbsgaKjabbccaGiabbQgaQjabbccaGiabbggaHjabbkhaYjabbwgaLjabbccaGiabbMgaPjabb6gaUjabbccaGiabbogaJjabb+gaVjabb6gaUjabbsha0jabbggaHjabbogaJjabbsha0bqaaiabicdaWaqaaiabb+gaVjabbsha0jabbIgaOjabbwgaLjabbkhaYjabbEha3jabbMgaPjabbohaZjabbwgaLbaaaiaawUhaaiaaxMaacaWLjaWaaeWaceaacqaIXaqmaiaawIcacaGLPaaaaaa@70CF@

We define two amino acids as being in contact if the distance between their *C*_*α *_is less than a given threshold.

XXStout predicts contacts at three different thresholds: 6 Å, 8 Å and 12 Å. Contact maps are predicted as follows: protein secondary structure, solvent accessibility and contact density are predicted from the sequence using, respectively, Porter, PaleAle and BrownAle; ensembles of two-dimensional Recursive Neural Networks [4,8,10] predict the contact maps based on the sequence, a 2-dimensional profile of amino-acid frequencies obtained from PSI-BLAST alignments of the sequence against the NR, and predicted secondary structure, solvent accessibility and contact density. XXStout is trained and tested in cross-validation on a sample of the S2171 dataset containing only sequences of length at most 200 residues (1602 proteins in total).

#### C_*α *_traces

3Distill is a server for the prediction of protein C_*α *_traces. 3Distill relies on a fast optimisation algorithm guided by a potential built on the 6 classes of structural features predicted by the other systems. A preliminary implementation of 3Distill (group Distill, ID 0348) [11], was ranked with model 1 (only one model was submitted) 9-th out of 181 predictors for GDT_TS on Novel Fold hard targets, and 20-th for Z-score for all Novel Fold and Near Novel Fold targets (13-th for NF-hard) at the CASP6 competition [20]. The online version currently available is a substantial improvement of the CASP6 one, based on more accurate structural feature predictions, and on a refined search algorithm. Homology information to structures in the PDB is also now exploited, when available. This is achieved by two means: secondary structure, solvent accessibility and contact maps are predicted by specialised systems which incorporate homology information as a further input [18]; structural similarity to homologues is directly used in the optimisation. Not surprisingly, the availability of homology information results in greatly increased accuracy of the models (see results section).

### Input format

**Figure 1:**
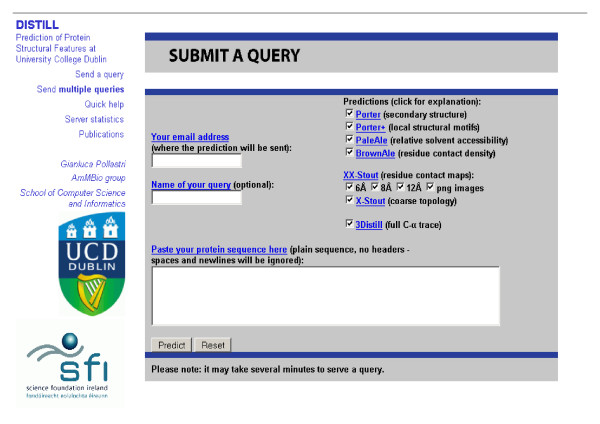
**Distill's single-query interface**. The multi-query interface is identical, except that the query name box is missing (names are extracted directly from the FASTA format).

Input into the servers is handled by two simple HTML forms: one for submissions of single queries (see Figure 1); the other for submitting multiple queries. In both forms the user must provide an email address, to which the response will be sent. In the single-query form the user has also the option of providing a query name, that will be reported in the server response. In the single-query form the user needs to provide the query sequence in plain 1-letter code, with no headers. New-line, tab and space characters are ignored. In the multi-query form the user may provide the queries in FASTA format, with each query name preceded by the > symbol on a line and the corresponding protein sequence on the following line or lines (space and tab characters are ignored). If the user adopts the multi-query form, a separate email will be sent for each query. The query name quoted in the response will be the query name as parsed from the FASTA format. In both forms the user can select any combination of the servers via a set of tick-boxes. Submissions of up to 32,768 characters (roughly 100 average proteins) are accepted by the multi-query form, making medium- to large-scale predictions possible with only a small number of manual submissions.

### Output format

Server responses are sent by email. One-dimensional feature predictions (secondary structure, structural motifs, solvent accessibility, contact density) are sent in plain text attached to the email. A detailed description of the codes, of their precise definitions, and an example of server response, are provided in the help web page [21]. The following is an example of server's response to a query sequence of length 106:

          Subject: Porter, PaleAle, BrownAle, XStout, XXStout, 3Distill,

          Porter+ response to 256BA

          Query_name: 256BA

          Query_length: 106

          Prediction:

          ADLEDNMETLNDNLKVIEKADNAAQVKDALTKMRAAALDAQKATPPKLEDKSPDSPEMKD

          CCCHHHHHHHHHHHHHHCCCCCHHHHHHHHHHHHHHHHHHHCCCCCCCCCCCCCCCHHHH

          bhHHHHHHHHHHHHHHgISHihHHHHHHHHHHHHHHHHHHgtCbEEEegsBTIihHHHHH

          EEbEEbeEebeEbbEeBeEbEebEeBeEBBEEBeEbBeeBbEbEbEEbEEeeEEbEebEe

          NNcNNccnnCcncCnnCccCccCccCCnCCccCCcCCCcCCnncNNNNNNNNNNNNncnc

          FRHGFDILVGQIDDALKLANEGKVKEAQAAAEQLKTTRNAYHQKYR

          HHHHHHHHHHHHHHHHHHHHCCCHHHHHHHHHHHHHHHHHHHHCCC

          HHHHHHHHHHHHHHHHHHHHHHHHHHHHHHHHHHHHHHHHHHHHhH

          bEEbbEebbebBeEBbEbBEEEebEEBeebBEEBeEbeEEbeEEbE

          CccCCccCCcCCCcCCcCCcccCCcCCCcCCccCcnccnnccnNnN

          Porter and PaleAle predictions based on PDB templates

          (seq. similarity up to 100.0%)

All one-dimensional features come predicted in 1-letter codes. The predictions are split into groups of lines of length 60. The first line in each group is the 1-letter code for the primary sequence (always present). The second line is the secondary structure predicted by Porter (always present). The symbols have the following meaning:

• H = helix: DSSP's H (*α*-helix) or G (3-10 helix) or I (*π*-helix) classes.

• E = strand : DSSP's E (extended strand) or B (*β*-bridge) classes.

• C = the rest : DSSP's T (turn) or S (bend) or . (the rest).

The third line represents predictions by Porter+. The line is present if the user asked for Porter+ predictions. Each residue is mapped into one of 14 possible structural motifs coded as 1-letter symbols – the 1-letter code for the motifs is devised to be mnemonically related to secondary structure (e.g. motifs 'H' and 'h' are frequent in helices, 'E' and 'e' in strands, etc.). The online help page provides a complete description of the codes representing Porter+ outputs.

The fourth line represents relative solvent accessibility predicted by PaleAle: B = completely buried (0–4% exposed), b = partly buried (4–25% exposed), e = partly exposed (25–50% exposed), E = completely exposed (50% exposed or more). The line is present if the user requested a PaleAle prediction.

The fifth line reports contact density predictions by BrownAle. Each letter in the sequence represents a contact density class: N = very low contact density (0,0.04), n = medium-low contact density [0.04,0.18), c = medium-high contact density [0.18,0.54), C = very high contact density [0.54, + ∞). This line is present if the user requested BrownAle predictions.

Two dimensional feature predictions (residue and coarse contact maps) come as files attached to the email. XStout's outputs come as 6 attachments:

• Attachment 1 (*number*.xstout4c, where *number *is a 5 digit code for the submission) : the distance map. The segments (as predicted by Porter – only helices and strand of length at least two are considered) are listed first, followed by one line for each pair of segments, indicating predicted distance range between the two contacts, followed by a reliability index. Distance ranges predicted are [0Å,10Å), [10Å,18Å), [18Å,29Å) and [29Å, + ∞). An example of XStout output is provided in the online help of the servers.

• Attachments 2–6 *(number.x*.topo.pdb, with *x *= 1 ... 5): 5 coarse reconstructions, in PDB format. Points represented are the termini of all Helices and Strands of length 2 or greater – there will be 2N such points in a protein with N segments.

**Figure 3:**
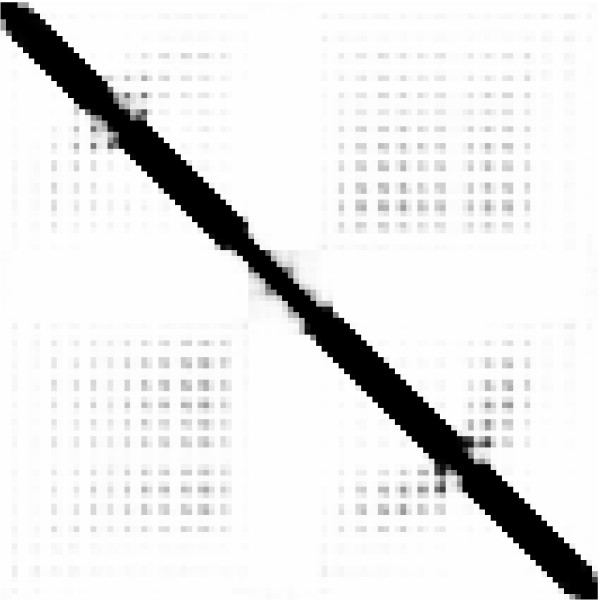
**XXStout PNG example output**. The 12Å contact map predicted for the example given in the "output format" section.

Residue contact maps defined at 6, 8 and 12 Å (files *number*.xxstout06, *number*.xxstout08 and *number*.xxstoutl2) are formatted as an N × N matrix of real numbers, where the *j*-th number on row *i *represents the estimated probability of contact between residues in positions *i *and *j*. PNG images containing grey-scale representations of residue contact maps are also automatically generated, if the user ticks the appropriate box. Figure 3 shows the PNG image of the contact map (thresholded at 12Å) for the previous example. Probabilities of contacts are represented as levels of grey ranging from pure black (certainty of contact) to pure white (certainty of non-contact).

If 3Distill is selected, 5 models for the C_*α *_trace are provided, if the query is at most 200 residues long. In case it is longer, fold predictions by XStout are automatically sent instead. The models come attached to the email in PDB format, and readable directly by common PDB viewers such as Rasmol [22]. An index of reliability of the model is provided in the remark fields of the PDB files. The index is an estimate of the TM score [23] of the model against the true structure, based on: the degree to which the model enforces the various predicted constraints; the size and estimated secondary structure composition of the query; the absence or presence (and, in the latter case, degree) of sequence similarity between the query and entries in the PDB. The reliability index, a novel component of Distill, is estimated by an artificial neural network trained in 5-fold cross-validation on 25800 C_*α *_trace reconstructions from the S258 set.

If homology information from the PDB is detected and used, this is indicated in the text of the response (alongside the percentage of sequence similarity to the best PDB template used) and in the remarks of the PDB files when these are present.

## Results

Porter, tested by a rigorous 5-fold cross validation procedure on S2171, achieves 79% correct classification on the "hard" CASP 3-class assignment (DSSP H, G, I → helix; E, B → strand; S, T, . → coil) [7], and currently has the highest performance (approximately 80%) of all servers tested by assessor EVA [24]. When homology information from the PDB is available, Porter's predictions are more reliable, up to over 90% correct (in the sense of matching DSSP assignments) when templates with over 90% sequence similarity are available [18], and about 88% for all residues for which any template information is available. This result is not surprising by itself because, although different programs for assigning secondary structure from the experimental structure often differ by up to 20%, once a semantics is chosen (e.g. DSSP over STRIDE or DEFINE) it is possible to classify secondary structure almost perfectly [25]. That is, it is true that there is some ambiguity in the assignment of secondary structures, but this is in large part due to the different definitions of secondary structures by different automated assignment programs, and only by a smaller amount to actual uncertainties as to what the structure may be.

PaleAle's accuracy, measured on the same large, non-redundant set adopted to train Porter (S2171) exceeds 53% correct 4-class classification, and roughly 80% 2-class classification (Buried vs Exposed, at 25% threshold). As in the case of Porter, predictive accuracy improves significantly when homology information is available [18], up to 70% correct prediction for the 4-class case and 87% for the 2-class one, when templates with over 90% sequence similarity are available, and roughly 65% for residues for which any kind of template information is available.

The accuracy of BrownAle, measured on S2171, is 46.5% for the 4-class problem, and roughly 73% if the 4 classes are mapped into 2 (dense vs. non dense). In both cases the classification performance of BrownAle is 16% above a base-line statistical predictor [4].

Tables 1 and 2 summarise the performances of XXStout measured on one fifth of the S2171 set after the exclusion of proteins longer than 200 residues (327 chains in total). Performances are given for the protein length/5 and protein length/2 contacts with the highest probability, for sequence separations of at least 6, at least 12, and at least 24, in CASP style [26]. These performances compare favourably with the best predictors at the latest CASP competition [4].

**Table 1:** XXStout performance.

separation	≥ 6	≥ 12	≥ 24
8Å	46.4% (5.9%)	35.4% (5.7%)	19.8% (4.6%)
12Å	89.9% (2.3%)	62.5% (2.0%)	49.9% (2.2%)

Top protein length/5 contacts classification performance as: precision%(recall%)

**Table 2:** XXStout performance.

separation	≥ 6	≥ 12	≥ 24
8Å	36.6% (11.8%)	27.0% (11.0%)	15.7% (9.3%)
12Å	85.5% (5.5%)	55.6% (4.6%)	43.8% (4.9%)

Top protein length/2 contacts classification performance as: precision%(recall%)

**Figure 4:**
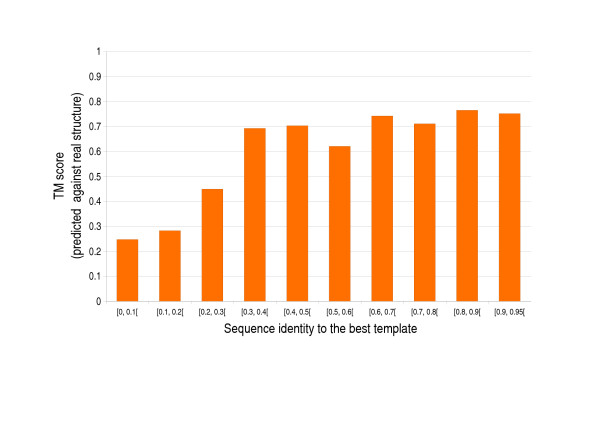
**Expected performances of 3Distill**. 3D reconstruction performances measured as average TM scores against the correct structure. Tested on the S258 set (see text for details). Maximum sequence similarity allowed to homologues in the PDB: 95%.

Figure 4 shows the expected accuracy of the C_*α *_trace reconstructor, as a function of sequence similarity to the closest homologue in the PDB, measured on the S258 set. The accuracy is measured as the TM score to the C_*α *_trace of the experimental structure. For sequence similarity above 30% the predictions' TM score is on average slightly above 0.7 indicating high reliability, is approximately 0.45 in the 20–30% interval, and 0.27 in the region below 20%. If reconstruction performances are measured on the S258 set without allowing homology information at any stage (pure *ab initio *predictions) the average TM score is 0.27, with 43 of the 258 structures above a TM score of 0.4 [11]. The reliability score reported in the remarks field of the PDB file has an average correlation of 0.7 with the TM score against the true structure, and thus provides a good estimate of the quality of the prediction.

## Conclusion

The servers we designed allow the annotation of protein sequences with a number of structural features which are at least partially orthogonal. Given the speed of the underlying methods, large- or even genomic-scale predictions can be handled by our servers in response of users' queries – with up to 32,768 residues handled in a single submission. Up to 20,000 queries per day can be processed by the servers based on their current implementation (a 40 CPU cluster), and nearly 30,000 tasks from 63 national or supernational domains have been served to date.

Our servers provide fast, reliable prediction of protein structural features for the *ab initio *case, and allow fast, reliable, large-scale predictions of protein structures for the case in which some homology to the PDB is detectable.

We are in the process of: extending the use of homology information to all prediction stages; building a parallel pipeline to Distill, for the case in which marginal similarity templates exist in the PDB for a query. Training of the systems for the latter case is completed and we expect that the new pipeline will be running over the next few months, complementing the current system with high-throughput fold recognition facilities.

## Availability and requirements

The servers are freely available for academic users at the address . Linux and Windows binaries for all the servers are freely available for academic users upon request. The sets used for training, testing and benchmarking the servers (S2171 and S258) are available upon request.

## Authors' contributions

DB designed implemented and benchmarked the C_*α *_trace reconstructor. AJMM designed and implemented the code for homology detection. CM designed and tested Porter+ and the homology-based versions of Porter and PaleAle. AV contributed the idea behind BrownAle, and created XStout. IW designed and implemented XXStout. GP designed Porter, BrownAle, and parts of XStout, and suggested the structure of the overall predictive pipeline, including its homology-based component. The manuscript was written by GP, AV and DB, and approved by all authors.
